# Characterization of a Novel Polymeric Bioflocculant Produced from Bacterial Utilization of *n*-Hexadecane and Its Application in Removal of Heavy Metals

**DOI:** 10.3389/fmicb.2017.00170

**Published:** 2017-02-07

**Authors:** Mihirjyoti Pathak, Hridip K. Sarma, Krishna G. Bhattacharyya, Sanjukta Subudhi, Varsha Bisht, Banwari Lal, Arundhuti Devi

**Affiliations:** ^1^Environmental Chemistry Laboratory, Resource Management and Environment Section, Life Science Division, Institute of Advanced Study in Science and TechnologyGuwahati, India; ^2^Department of Biotechnology, Gauhati UniversityGuwahati, India; ^3^Department of Chemistry, Gauhati UniversityGuwahati, India; ^4^Environmental and Industrial Biotechnology Division, The Energy and Resources InstituteNew Delhi, India

**Keywords:** *n*-hexadecane, bioflocculant, contact angle analysis, removal of heavy metals, glycoprotein, bioremediation

## Abstract

A novel polymeric bioflocculant was produced by a bacterium utilizing degradation of *n*-hexadecane as the energy source. The bioflocculant was produced with a bioflocculating activity of 87.8%. The hydrocarbon degradation was confirmed by gas chromatography-mass spectrometry analysis and was further supported with contact angle measurements for the changes in hydrophobic nature of the culture medium. A specific aerobic degradation pathway followed by the bacterium during the bioflocculant production and hydrocarbon utilization process has been proposed. FT-IR, SEM-EDX, LC/MS, and ^1^H NMR measurements indicated the presence of carbohydrates and proteins as the major components of the bioflocculant. The bioflocculant was characterized for its carbohydrate monomer constituents and its practical applicability was established for removing the heavy metals (Ni^2+^, Zn^2+^, Cd^2+^, Cu^2+^, and Pb^2+^) from aqueous solutions at concentrations of 1–50 mg L^-1^. The highest activity of the bioflocculant was observed with Ni^2+^ with 79.29 ± 0.12% bioflocculation efficiency.

## Introduction

The applicability of microorganisms for biodegradation of hazardous substances, such as xenobiotics, pesticides or even crude petroleum has been a subject of interest due to the wide range of possible uses in various bioremediation procedures and may be called as a “green process.” Attention is particularly directed toward isolating robust microbes from nature which could be employed for remediation of pollutants as chemical agents are found effective in degradation but may lead to various toxicity issues. Assessment of microbes for their remediation potential in dealing with industrial pollutants is another point of interest where a bacterium or a fungus produces metabolites as their weapon of degradation. An important area of increasing interest in this is to investigate novel metabolites or macromolecules generated by microbes during the process of metabolism. Researchers are also taking interest to selectively investigate such metabolites for their use in treatment of environmental pollutants including waste water pollutants containing heavy metals contaminants generated from various refineries, industries, and mills (Barakat, 2011). Currently the industries have up taken various strategies for removal of organic and metallic contaminants from waste water, such as sedimentation, membrane filtration, coagulation-flocculation, adsorption, chemical precipitation, and ion-exchange, etc. (Aracic et al., 2015). In this respect, the remediation of industrial waste water with a large input of variety of toxic heavy metals, has received considerable attention with wide-ranging interests in “bioflocculation” procedures that utilize an active metabolite, the “bioflocculant” in the process.

The bioflocculants are environment-friendly biological macromolecules and have been tested successfully for treating wastewater. There have been several classes of bioflocculants originating either naturally or prepared with cost-effective nutrients. Bioflocculant production by microbes utilizing a variety of media like brewery wastewater, soybean juice, fishmeal wastewater, etc., has been reported in the literature (Zhang et al., 2007). Microbes produce various polysaccharides that are classified by their biological functions into intracellular storage polysaccharides, capsular polysaccharides (closely linked to the cell surface) and extracellular polysaccharides (such as xanthan, sphingan, alginate, cellulose, etc.). Bacteria produce a wide range of exopolysaccharides which are synthesized via different biosynthesis pathways (Jochen et al., 2015). These polysaccharides play an important role in biofilm formation (Schmid and Sieber, 2015). The extracellular polysaccharides are biodegradable in nature and are notably identified as exopolysaccharides (EPS), glycoproteins, glycolipids, etc. (Lian et al., 2008). The EPS is likely to play a critical role in biosorption of metals. The extracellular metabolites excreted by bacteria and fungi or produced from cell lysis, might have some flocculation activity and might play the role of an energy source for microbes during starvation, as mediator in cell–cell interactions, facilitating the adherence of cells to the surface. Thus, they could become instrumental in biofilm formation and in microbial aggregation (Shi-Jie et al., 2011). Recent investigations suggest that the activity of exopolysaccharides in biofilm formation may be responsible for making the bacterium comfortable to a hydrophobic substrate such as phenanthrene, a high molecular weight PAH (Mangwani et al., 2014).

Interestingly, in the production of bioflocculants, the microbes can utilize unusual carbon sources as nutrient along with the production of other bacterial metabolites. It has been reported that a *Rhodococcus* could have efficiently utilized alkanes in biosurfactant or bioflocculant production and in the process, could have degraded to linear and branched alkanes (Sayavedra-Soto et al., 2006). Recent studies show the potential of a known petroleum hydrocarbon scavenger microbe to produce bioflocculants during its metabolic process (Pathak et al., 2015). In the present work, crude petroleum (the so-called “sweet crude” from Assam oil fields, India with abundant aliphatics and PAHs in it) itself has been taken as the hydrocarbon source. An attempt has been made to observe the particular behavior of a hydrocarbon degrading microorganism that is not amended with typical mineral medium. It is thought that the study will be able to find a few answers to the chemistry behind production of bioflocculant.

Studies reported from the same laboratory have shown that the subject bacterium is capable of degrading crude oil that contains as many as 34 different petroleum hydrocarbons (Pathak et al., 2015). However, the main objective behind the present study is to understand whether the bioflocculant production by the bacterium is dependent on the complex mixture of hydrocarbons in the crude oil as a whole or was dependant on a particular hydrocarbon only for displaying bioflocculating property, or could the activity be enhanced by any of the hydrocarbons present in the crude petroleum in producing one or more of the bioflocculants. Thus, this study serves as a model in investigating some of the unanswered questions with respect to utilization of a single petroleum hydrocarbon substrate and generation of a bioflocculant to be utilized as an alternative to typical chemicals for removing heavy metals from the environment.

## Materials and Methods

### Chemicals

All the chemicals, standards, and petroleum hydrocarbons were purchased from HiMedia chemicals, MERCK, and Sigma-Aldrich.

### Bacterial Utilization of Hydrocarbons

A potent bioflocculant producing bacterium, capable of catabolizing the hydrocarbons in crude petroleum was selected for this work. This bacterium was identified as *Pseudomonas aeruginosa* strain IASST201 by using 16srDNA sequencing technique and the sequence was submitted to GenBank with accession number KF583972. It exhibited bioflocculating activity highest among other bacterial isolates screened from activated sludge samples collected from Numaligarh Refinery Limited, Bongaigaon Refinery Limited and Noonmati Refinery of the Indian Oil Corporation Limited, Assam, India. Data published earlier from this laboratory (Pathak et al., 2015) have confirmed the presence of 34 different petroleum hydrocarbons in the tested crude oil which was to be utilized as the only carbon source by the bacterium to produce bioflocculant *in vitro* in an optimized production medium. In the present work, crude oil in the same optimized production medium was initially replaced with 1% mixture of 34 petroleum hydrocarbons. The initial pH of the medium was maintained at 7.2 and the same was incubated in an orbital shaker at 37 ± 2°C with a rotation speed of 150 rev min^-1^. For estimating bioflocculant activity, the methodology suggested by Kurane et al. (1986) was adopted. After the incubation period was complete, the bacterial culture grown supernatants were obtained by centrifuging the culture broth at an rpm of 6000 maintaining the temperature at 4°C. This was used as raw bioflocculant for initial evaluation. The bioflocculating activities of each isolate were determined by using the culture broth supernatant as raw bioflocculant. 0.04 g of kaolin clay was suspended in 9.45 mL distilled water, mixed thoroughly with 0.5 mL of 1% CaCl_2_ solution. 0.05 mL of the culture supernatant was then added to the suspension and the pH was adjusted to 7.0 with very dilute NaOH and HCl. The mixture suspension was vortexed in a test tube for 1 min and kept for 5 min at room temperature. The bioflocculating activity was determined using the formula:

$$\text{Bioflocculating activity }=\left[\left({\text{A}}_{\text{s}}-{\text{A}}_{550}\right)/{\text{A}}_{s}\right]\times 100\%$$

where, A_550_ is the absorbance of the supernatant while A_s_ is the absorbance of the blank without any prior treatment. Absorbance was read at 550 nm in a UV-VIS spectrophotometer (Shimadzu 1601, Japan).

The screening of bioflocculating activity of each hydrocarbon amended broth was determined till seventh day of inoculation at 24 h intervals. The optimum pH (5–9) and the concentrations (0.1–5.0%) of the hydrocarbons necessary to obtain the highest bioflocculating activity was determined as well. The growth of bacteria in the production medium amended with single hydrocarbon was verified by measuring the optical density of the bacterial broth at 600 nm.

### Production and Purification of Bioflocculant from Suitable Carbon Source

The most effective hydrocarbon source supporting growth of the bacterium was determined on the basis of highest bioflocculating activity exhibited by the bacterium during its utilization *in vitro*. Increase of bioflocculating activity in the culture medium was observed along with bacterial growth and the growth vs. bioflocculating activity curve was constructed. This helped in mass production of the bioflocculant under optimized parameters. The bioflocculant produced by the strain IASST201, was extracted from the production broth supernatant (6000 ×*g* for 30 min) by ice-cold ethanolic precipitation and then kept for 12 h to separate the bioflocculant content. The resulting precipitate was collected by centrifugation, lyophilized and solubilized with deionized water (conc. 10 mg mL^-1^) and purified with a DEAE-cellulose-52 column. The purified bioflocculant was eluted with deionized water and a grade of (0.1–1 M) NaCl in phosphate buffer (pH 7) maintaining a flow rate of 0.5 mL per min (Wang et al., 2014).

### Characterization of the Bioflocculant

The protein concentration of the bioflocculant was measured by the Bradford method (Bradford, 1976) and the total carbohydrate content was determined with the Anthrone method (Gerhardt et al., 1994). The biochemical assays were used to determine the presence of different sugars. The Purified bioflocculant was further subjected to FTIR in ATR mode (500–4000 cm^-1^).

The purified bioflocculant from the DEAE column was lyophilized and was subjected to FE-SEM. The scanning electron microscopic images were taken at 5 kV with a FE-SEM (Zeiss, P-Sigma, Germany). The EDX measurements were performed with an X-ray detector and were analyzed with INCA 4.15 EDS software (Oxford Instruments). The same was subjected to PerkinElmer thermogravimetric analysis (TGA) 4000 at the heating rate 10°C/min and with a nitrogen flow rate of 20 mL/min.

The protein content of the bioflocculant was separated by chloroform: butyl alcohol (5:1) extraction. The protein-free bioflocculant was dialyzed against de-ionized water overnight, vacuum-dried, followed by hydrolysis with 2N trifluoroacetic acid (TFA) in a hydrolysis tube (Neeser and Schweizer, 1984). Excessive TFA was removed, denatured protein debris were precipitated with trichloroacetic acid, followed by incubation in ice for 30 min and further centrifugation (10000 ×*g* for 30 min at 4°C). EPS was precipitated by ethanolic precipitation and the pellets were obtained through centrifugation (10000 ×*g* for 30 min at 4°C). It was then reconstituted with 1 mL of acetonitrile/water (80:20 v/v) and analyzed for different structural analogs by LC/MS (1260 Infinity LC fitted with 6410 Triple Quadrupole MS, Agilent Technologies, USA). A 2 μL sample aliquot was injected into a ZORBAX C18 column using a gradient of water + 0.1% formic acid (solvent A) and acetonitrile (solvent B) at 40°C maintaining a flow rate of 0.2 mL min^-1^ with modifications (François et al., 2012). ESI-MS spectra were obtained in positive ion mode and were analyzed using Agilent ChemStation Software. Full scan data were obtained by scanning from *m/z* ratio of 50–950 with a fragmentor voltage calibrated at 135.0 V.

For nuclear magnetic resonance (NMR) spectroscopic analysis, the column purified bioflocculant samples were prepared by dissolving purified bioflocculant (5 mg) into 1 mL of D_2_O and shaking it overnight at 50 ×*g*. This solution was then transferred into the 5-mL NMR tube, capped and subjected to ^1^H NMR (400 MHz, Varian-AS400) following a modified method (Nwodo and Okoh, 2012).

### Characterization of the EPS Monomers

For identification of EPS monomers of the bioflocculant, gas chromatography-mass spectrometry (GC/MS) was done after derivatization of the non-volatile sugar monomers to a compatible volatile state. Dried EPS residue was dissolved in 80 μl of 20 mg mL^-1^ methoxyamine hydrochloride in pyridine for 90 min for 30°C. This was further derivatized by adding 80 μl of *N*-methyl-*N*-(trimethylsilyl) trifluoroacetamide (MSTFA) and incubated for 30 min/37°C. Chromatography was done with an EB-5MS column (Agilent, USA) attached in a triple quadruple GC/MS (TQ8030, Shimadzu, Japan) supported with GC/MS solution software (version 4). Helium was used as the carrier gas with a flow rate of 1.0 mL min^-1^. GC/MS analysis was carried out with a modified method (Roessner et al., 2000). Mass range (*m/z*) was selected from 45 to 800 for the entire analysis. The GC program was optimized for detection of derivatives and all analyses were carried out with the split ratio of 20:1.

### Study of Hydrocarbon Utilization

In order to determine the utilization of the most effective hydrocarbon in production of the bioflocculant by the selected bacteria, the optimized production media broth was extracted (v/v) with dichloromethane (DCM) when the bioflocculating activity was found highest (Esin et al., 2011). The DCM extracted proportion of the hydrocarbon (control) and degraded portion (test) were both subjected for FTIR analysis. Furthermore, the DCM extracted proportion of the control and the test were analyzed though a GC/MS (TQ8030, Shimadzu, Japan) with an autoinjector (AOC 20I, GC-2010, E) to obtain the degradation percentage as well as the degradative intermediates. The split ratio was set at 20:1. Helium was used as the carrier gas with a flow rate of 1.0 mL min^-1^. Injection temperature was set at 300°C. The column and oven temperatures were set at 60°C with a hold time of 5 min and were subsequently increased to 300°C with a ramp of 10°C min^-1^. The final hold maintained was for 44 min. The ion-source temperature was set at 230°C for MS using an interface temperature of 310°C. Mass range (*m/z*) was selected from 45 to 800. For compound identification, NIST 11 library database was used. The percentage degradation of the hydrocarbon was calculated by the formula, [(mHc - mH)/mHc] × 100, where mHc and mH were the sum of the total areas of peaks for the control sample and the test samples, respectively (Pathak et al., 2015).

To test the changes in hydrophobic nature of the *n*-hexadecane amended broth due time, the grown culture broth was initially made cell free and then were subjected to contact angle (CA) measurements against a microscope glass slide (76 × 26 × 1 mm) with a release amount of 10 μl through automatic syringe and was analyzed with a CA analyzer (DSA30 KRÜSS, Germany).

### Heavy Metal Removal by Bioflocculant

The bacterial bioflocculant produced and purified from the culture grown broth after the single hydrocarbon degradation was employed for the bioflocculation test against aqueous solutions containing Ni^2+^, Zn^2+^, Cd^2+^, Cu^2+^, and Pb^2+^ at concentrations 1, 10, 20, 30, 40, and 50 mg L^-1^, respectively. The bioflocculation experiment was performed with both the raw bacterial culture supernatant and the purified bioflocculant. The metal concentrations in the upper layer of the solution were measured with an Atomic Absorption Spectrometer (Shimadzu AA7000, Japan). The instrumental conditions applied in the detection of heavy metals were given in **Table 1**.

**Table 1:** Instrumental conditions used for estimation of heavy metal concentrations through AAS.

	AAS instrumental conditions				
Metals	Wave length (nm)	Slit (nm)	Flame type	Flow (L min^-1^)
Ni	232.0	0.2	Air-C_2_H_2_	1.6
Zn	213.9	0.7	Air-C_2_H_2_	2.0
Cd	228.8	0.7	Air-C_2_H_2_	1.8
Cu	324.8	0.7	Air-C_2_H_2_	1.8
Pb	283.3	0.7	Air-C_2_H_2_	2.0

**FIGURE 1:**
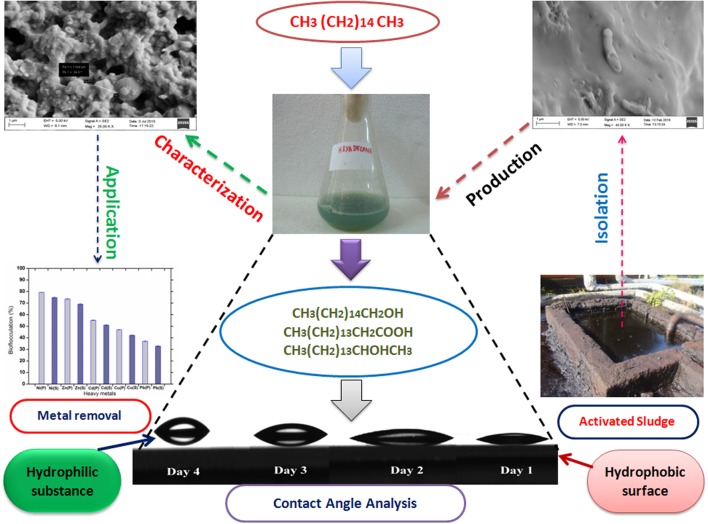
**A schematic representation of practical application of a bioflocculant synthesized by a bacterium isolated from hazardous environment and its “greener” action toward bioremediation observed through novel analytical tool like CA**.

The whole scheme of study toward remediation of heavy metals through a bioflocculant synthesized by utilizing a hydrocarbon source is presented with a schematic artwork as **Figure 1**.

### Software and Statistics

All the experiments were performed in triplicates and the error bars in the figures represent the standard deviations of the data. The entire data analyses were performed in Origin (version 8.5). For construction of comparative graphs from FTIR-ATR data OMNIC version8 software was used. For compound drawing ChemDraw Ultra 8.0 and for identification through GC/MS, NIST 11 library database was used.

## Results

### Utilization of Single Hydrocarbons by the Selected Bacterium

Preliminary investigations indicated that 2% crude oil was optimum for the better display of bioflocculating activity in the production medium with peptone and CaCO_3_ as optimum nitrogen and cation source as reported earlier (Pathak et al., 2015). Whilst singly amended petroleum hydrocarbons in the medium revealed interesting information about bioflocculant production by this bacterium. The optimization conditions for time, pH and concentrations for each hydrocarbon present in crude oil were maintained to identify the most potential single hydrocarbon source responsible for furnishing higher flocculating activity (**Table 2**).

**Table 2:** The utilization of *n*-hexadecane at best to produce the efficient bioflocculant when compared with the utilization data of all the petroleum hydrocarbons detected in DCM extracted portion of the subject crude oil.

Hydrocarbons	Time of highest activity (h)	Optimum pH	Optimum concentratio*n* (%)	Bioflocculating activity (%)
**Aliphatic hydrocarbons**				
Undecane	48	6	0.5	50.78 @ 0.009
Tridecane	72	6	1.0	64 @ 0.10


Tetradecane	72	6.5	0.5	69.10 @ 0.08


Pentadecane	96	6	1.0	58.4 @ 0.21


Hexadecane	120	7	1.0	87.8 @ 0.02


Heptadecane	72	7	0.5	63.75 @ 0.02


Octadecane	96	6.5	0.75	68.96 @ 0.01


Nonadecane	72	6	0.25	47.5 @ 0.06


Dodecane	96	6	0.5	73.85 @ 0.03


Eicosane	96	7.5	0.75	54.27 @ 0.02
Heneicosane	96	7.5	0.25	43.21 @ 0.03
Tricosane	96	8	0.25	42.78 @ 0.11
Tetracosane	72	8	0.50	52.21 @ 0.14
Pentacosane	96	8	0.25	38.96 @ 0.02
Hexacosane	72	8	0.25	36.14 @ 0.02
Heptacosane	96	8	0.25	33.21 @ 0.02
Octacosane	96	8	0.25	32.14 @ 0.03
Nonacosane	72	7.5	0.25	29.28 @ 0.04
Docosane	72	7.5	0.50	67.21 @ 0.05
Triacontane	72	6.5	0.50	41.85 @ 0.02
Hentriacontane	96	6.5	0.25	13.92 @ 0.034
Dotriacontane	96	6.5	0.50	48.53 @ 0.03
Tritriacontane	72	6	0.50	40.89 @ 0.056
Tetratricontane	96	6	0.25	33.57 @ 0.025
Pentatricontane	96	6	0.25	32.85 @ 0.051
Hextriacontane	96	6	0.25	30.35 @ 0.03
Heptatriacontane	48	6	0.25	28.67 @ 0.015
Pristane	72	7	0.25	18.21 @ 0.02
Phytane	96	7	0.75	24.48 @ 0.025
**Aromatic hydrocarbons**				
Benzene	72	6	0.5	52.82 @ 0.017
Indene	72	6.5	0.25	31.07 @ 0.015
**Polyaromatic hydrocarbons**				
Naphthalene	72	6.5	0.50	64.85 @ 0.03
Phenanthrene	48	6	0.50	52.17 @ 0.015
Fluorene	72	6	0.25	39.03 @ 0.02

**FIGURE 2:**
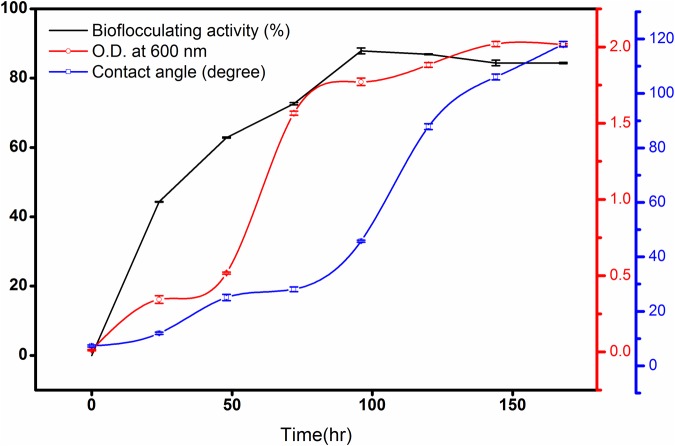
**Bioflocculating activity of the bacteria against time showing the rise of contact angle (CA) of the broth against a hydrophobic surface**. The error bars presents the ±SD of the data.

In this case, *n*-hexadecane was found to be the most effective single hydrocarbon throughout the experimentations that exhibited highest flocculating activity as high as 87.8% at the 96^th^ hour of incubation with a bioflocculant yield of 1.98 g L^-1^ in the optimized production medium. Alternatively, the bacterial growth curve entered the stationary phase by 144th hour of incubation and this was followed by gradual decrease in bioflocculation (**Figure 2**).

### Analysis of *n*-Hexadecane Degradation

**FIGURE 3:**
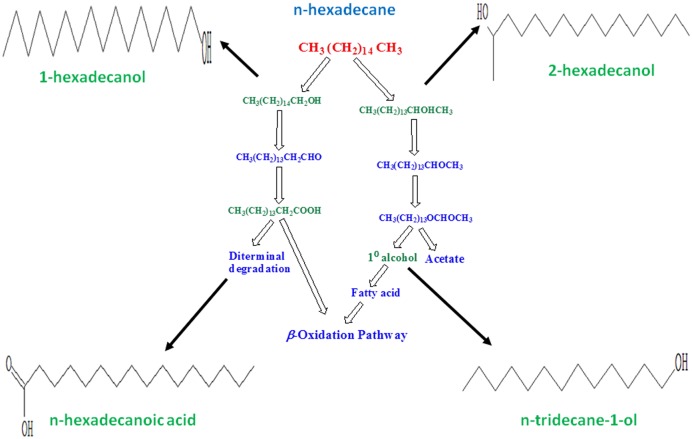
**Figure showing possible pathway followed by selected bacterium in utilizing *n*-hexadecane as substrate**. Degradation intermediates were identified through GC/MS study as n-tridecane-1-ol, 2-hexadecanol, n-hexadecanoic acid and 1-hexadecanol. Molecular structures in this figure indicate the respective compounds detected through GC/MS analysis.

Certain bacterial species, referred as hydrocarbonoclastic bacteria, are reported to be highly specialized in degrading hydrocarbons. They play a key role in the removal of hydrocarbons from polluted environments by typically utilizing aliphatic hydrocarbons through oxidative processes (Singh et al., 2012). The GC/MS analyses which indicated degradation of *n*-hexadecane by 83.44% in the bacterial broth, when the bioflocculating activity was found highest. In the degraded sample, n-tridecane-1-ol, 2-hexadecanol, n-hexadecanoic acid, and 1-hexadecanol were detected at retention times 15.780, 20.853, 22.670, and 22.992 min, respectively. The degradation of *n*-hexadecane indicated that single and less complex hydrocarbons could be easily utilized by the specific bacterium as compared to complex mixture of aliphatic and aromatic hydrocarbons in crude petroleum oil. An approach to correlate the by-products of the subject n-alkane degradation with microbial production of bioflocculant as metabolite is described here which was evident from the GC/MS based study (**Figure 3**).

### Changes in Hydrophobicity during Bioflocculant Production

As biodegradation proceeds till the maximum bioflocculating activity, the CA increased rapidly as 7.3 ± 0.4, 11.9 ± 0.4, 25.1 ± 1.15, 28.1 ± 0.85, 45.7 ± 0.38, 87.8 ± 1.05, 106.03 ± 1.05, and 118.1 ± 1.0 degree (**Figure 2**) against the hydrophobic glass plate. This indicates increasing hydrophilic nature of the degradation intermediates formed gradually in the culture medium The measurements, based on CA measurement, in following the degradation kinetics of a bacterium in converting a hydrocarbon like *n*-hexadecane to more hydrophilic fragments, can be considered as a completely newer approach in studying degradation of a hydrophobic substance in liquid medium.

### Biochemical, FTIR, SEM-EDX, TGA, and ^1^H NMR Characterization of Bioflocculant

**FIGURE 4:**
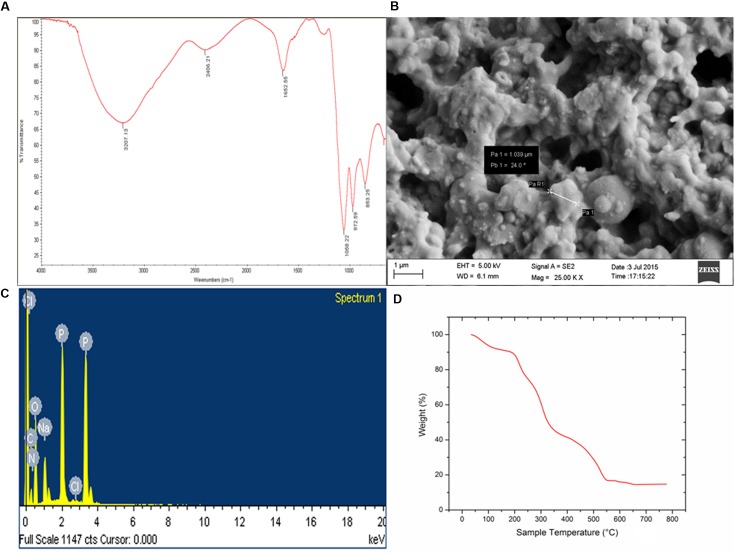
**(A)** FTIR-ATR spectra of purified bioflocculant showing presence of hydroxyl, amino, and carboxylic group. **(B)** Irregular shape of the extracellular bacterial bioflocculant. **(C)** SEM-EDX spectra depicting presence of C, N, O, Na, P, and Cl in the purified bioflocculant content. **(D)** TGA showed stability of carbohydrate and protein portion in bioflocculant.

Biochemical tests of the column purified bioflocculant indicated it to be a composition of sugar and protein in the proportion 89.4 and 6.2%, respectively and furthermore confirmed the presence of reducing sugars, amino sugars and uronic acids. In present case, FTIR spectra became indicative of the presence of functional groups related to sugars and proteins (**Figure 4A**). A strong absorption peak was observed at 3207 cm^-1^ generated by –OH or –NH stretching vibrations, and a weak C–H stretching band was found at 2406.21 cm^-1^. Absorption peak at 1652.55 cm^-1^ defined the presence of carbonyl group indicating characteristic vibrations of the C=O stretching in the –CONH– group in proteins and amino-sugars (Sun et al., 2015a). The wave numbers from 1200 to 800 cm^-1^ is significant different polysaccharides (Copikova et al., 2006). Bands at 1058.22 cm^-1^ are attributed to asymmetrical stretching of the C–O–C ester linkage while the presence of *β*-glycosidic linkages between the sugar monomers is indicated by a small absorption band at 861.82 cm^-1^.

The elemental analysis of the column purified bioflocculant on the bais of SEM-EDX experiment revealed presence of C, N, O, Na, P, and Cl in this macromolecule as 31.20, 6.12, 46.39, 5.67, 10.40, and 0.23%, respectively (**Figures 4B,C**). The SEM image showed the bacterial bioflocculant to be amorphous and irregularly shaped biopolymer.

The TGA analysis of the purified bioflocculant indicated presence of carbohydrate-protein polymer on the basis of thermal stability. Interestingly the weight/temperature curve lowers till ∼222°C but the weight was not lost more than 20% and the gradual fall of curve depicted presence of highly thermostable content (viz., carbohydrate) and a less thermo-stable content (viz., protein) in the bioflocculant constitution (**Figure 4D**). The first weight loss what was observed in this case could be due to the loss of moisture content in the bioflocculant or the protein part which is associated with glycoprotein-like bioflocculant. However the weight loss of the sample is constant from ∼550°C up to end of the run indicating the strong carbohydrate backbone of the bioflocculant.

**FIGURE 5:**
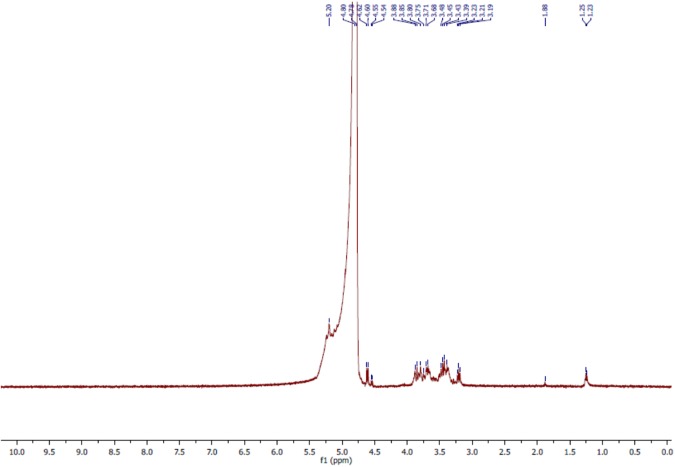
**^1^H NMR spectra of purified bioflocculant indicated presence of nonanomeric proton, -NH group, carbonyl and CH_2_ linkages**.

The ^1^H NMR analyses of the bioflocculant significantly reflected the results obtained from FTIR study. The spectral analysis of bioflocculant (**Figure 5**) revealed the presence of signal at 5.2 ppm, shows the presence of -NH group. The solvent peak came at around 4.8 ppm. Again, signals at 4.60–4.62, 3.19–3.88, and 1.23–1.25 ppm confirmed the presence of hydroxyl proton, non-anomeric protons and CH_2_ linkage in the sample, respectively. Again, a peak around 1.88 ppm indicated the presence of carbonyl linkage of glycoprotein chains. These results ascertained the composition of bioflocculant to be of carbohydrate and proteinous substance or as of glycoprotein polymeric-origin.

### Mass Spectrometry Based Characterization of Bioflocculant EPS

**FIGURE 6:**
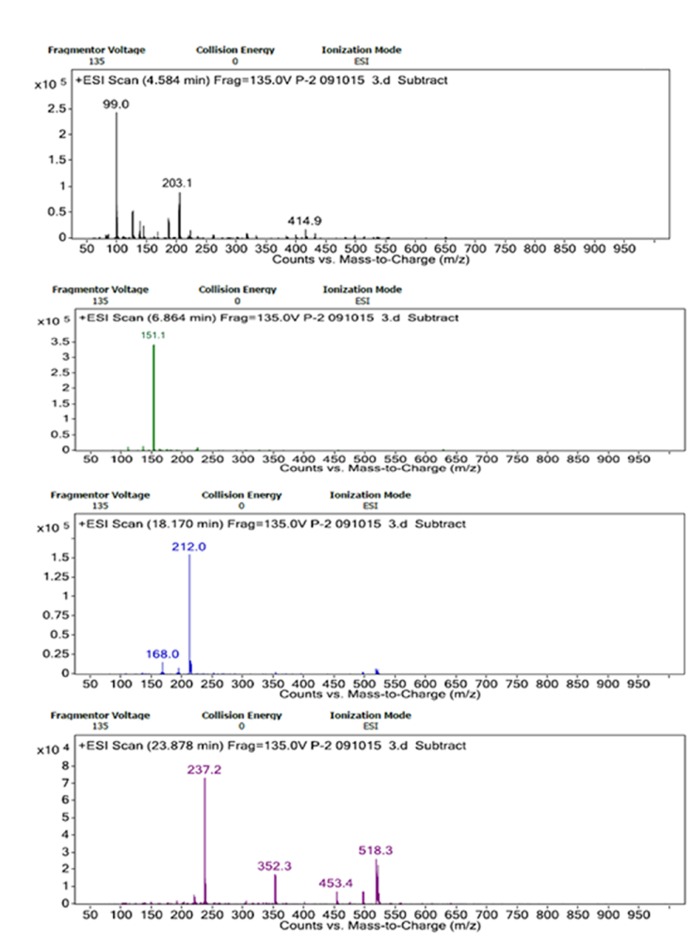
**ESI spectra of LC/MS analyses of hydrolyzed bacterial bioflocculant showing Na^+^, H^+^, and NH_4_^+^ adducts of glucose, xylose, glucuronic acid, and rhamnose. (A–D)** showed elution of these bioflocculant monomers at different retention time of LC/MS respectively.

Through the LC/MS based analysis, adducts of Na^+^, NH_4_^+^ and H^+^ ions were identified for the sugar monomers depending on fragmentation and elution time in present case. Na^+^ adducts for glucose appeared as (Glc+Na)^+^ (*m/z* 203.1) and rhamnose as (Rha+Rha+Na)^+^ (*m/z* 352.3) at retention time 4.584 and 23.878 min, respectively. The *m/z* ratio of 212.0 represents glucuronic acid may forming adduct as (GlcA+NH_4_)^+^ at 18.170 min. The presence of xylose could be predicted from the formation of proton adducts with *m/z* of 151.1 (Xyl+H)^+^ at 6.864 min (**Figures 6A–D**).

**FIGURE 7:**
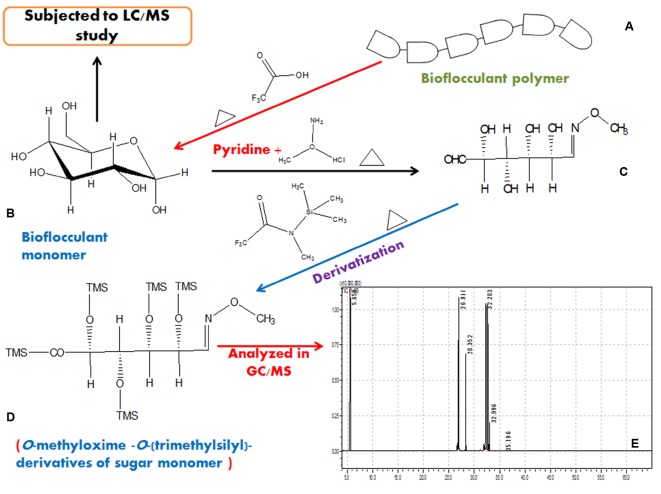
**A representation of hydrolysis of bioflocculant and characterization in GC/MS after proper derivatization in steps**. A typical bioflocculant polymer **(A)** was hydrolyzed to obtain monomers **(B)**, then reduced **(C)**, derivatized **(D)**, and subjected to GC/MS analysis **(E)**.

Together with these, from the GC/MS analysis of derivatized bioflocculant sample a number of sugar monomers, viz., D-Xylose, L-Rhamnose, D-Glucose, D-glucuronic acid and traces of *N*-acetyl D-glucosamine (retention times of 26.941, 28.352, 32.283, 32.996, and 35.796 min, respectively) was detected and compared with standard sugars. The solvent peak appeared at 5.659 min. These results indicate that the bioflocculant consists of five basic sugar monomers. The step-by-step scheme of GC/MS based characterization of the EPS is presented in **Figure 7**.

### Application of Bioflocculant for Removal of Heavy Metals

**FIGURE 8:**
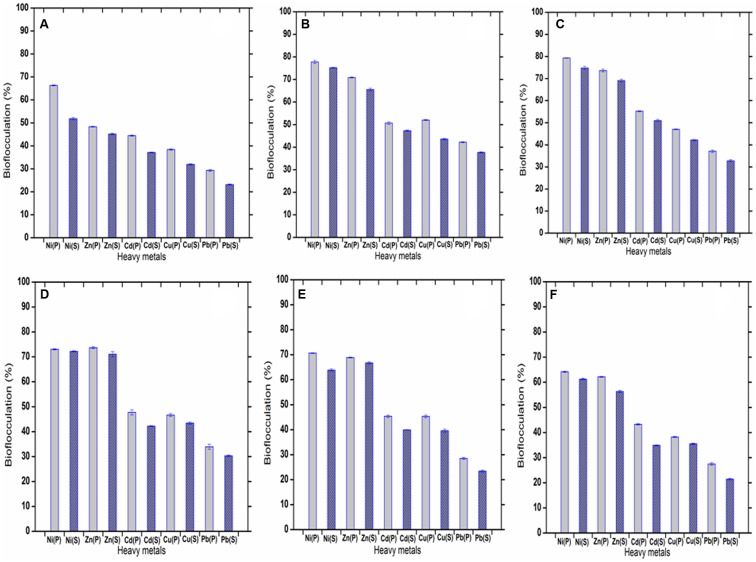
**(A–F)** showing flocculation of five heavy metals varied in concentration of 1, 10, 20, 30, 40, and 50 mg L^-1^. (P) and (S) defined the purified and raw bioflocculant, respectively.

The metal ions, Ni^2+^, Zn^2+^, Cd^2+^, Cu^2+^, Pb^2+^ within a range of 1–50 mg L^-1^ in aqueous solutions could be successfully settled by the bioflocculant (highest removal of Ni^2+^, Zn^2+^, Cd^2+^, Cu^2+^, Pb^2+^ was possible at concentrations of 20, 30, 20, 10, and 20 mg L^-1^, respectively). The flocculation rate achieved with the purified bioflocculant was 79.29 ± 0.12, 73.7 ± 0.4, 55.21 ± 0.24, 52 ± 0.18, and 42.21 ± 0.18%, respectively for Ni^2+^, Zn^2+^, Cd^2+^, Cu^2+^, Pb^2+^ with the efficiency of removal being in the order of Ni^2+^ > Zn^2+^ > Cd^2+^ > Cu^2+^ > Pb^2+^ at the optimum pH of 7.0. The bioflocculation caused by the supernatant bioflocculant (without column purification) was 78.18 ± 0.16, 71.06 ± 1.01, 51.03 ± 0.45, 43.57 ± 0.34, and 37.66 ± 0.23%, respectively for Ni^2+^, Zn^2+^, Cd^2+^, Cu^2+^, Pb^2+^ (highest in aqueous solutions of 20, 30, 20, 10, and 10 mg L^-1^, respectively) with the same pattern of efficiency as Ni^2+^ > Zn^2+^ > Cd^2+^ > Cu^2+^ > Pb^2+^. When the purified and the supernatant bioflocculants are compared with respect to their metal-flocculating capacities, the purified one was observed to be a little more efficient (**Figure 8**). The difference might be due to the state of the bioflocculant surface. It may be noted that the electronic properties of the metals and the physicochemical status of the biological material determine the metal-removal capacity. In case of the bioflocculant, active functional groups like carboxyl, amino, and hydroxyl groups on the surface might also be an important factor (Khakimov et al., 2013).

## Discussion

The bacterium, *P. aeruginosa* strain IASST201 dwelling in activated sludge of refinery was investigated for its activity against different hydrocarbon amended media. Already this isolate was reported for its peculiar characteristics of utilizing crude petroleum oil for production of efficient bioflocculant by our team (Pathak et al., 2015). However, the concentration of petroleum hydrocarbons in a complex nutrient source like crude oil is highly intrusive and the responsible hydrocarbon acting as the active carbon source triggering the bacteria to produce bioflocculant was not appreciated in earlier reports. It was pertinent that a deeper insight into defragmenting the conditions of hydrocarbons distributed in the crude oil composition was inevitable to understand the dependency of the bacteria for its sustenance and production of bioflocculant.

Aerobic alkane degraders often activate alkane molecules using O_2_ as a reactant. It is also known that alkane-activating monooxygenase overcome the low reactivity of hydrocarbons by producing reactive oxygen species. In the case of methyl oxidation by some microbes, degradation is initiated by the oxidation of a terminal methyl group to a primary alcohol, then oxidized to aldehyde and finally to a fatty acid. These in turn get processed by the *β*-oxidation pathway. The degradation intermediates shown in **Figure 3** relate to the present microbial action along the aerobic degradation pathway of alkane mentioned by Fritsche and Hofrichter (2000). **Figure 3** shows how possibly a typical aliphatic hydrocarbon may be converted to simpler molecules through microbial actions. In this, the molecular structures were drawn for n-tridecane-1-ol, 2-hexadecanol, n-hexadecanoic acid, and 1-hexadecanol, respectively. These compounds have been detected through GC/MS analysis. The presence of these compounds indicates the degradation of *n*-hexadecane to simpler molecules during its utilization by the *Pseudomonas* species. An aerobic degradation pathway is suggested. The metabolic process caused by the bacterium converts the target hydrocarbon (*n*-hexadecane) to primary or secondary alcohol species at different cleavage points of the carbon chain, also leading to formation of aldehydes with less number of C-atoms than the target compound. Acids generated by diterminal degradation were also detected in the degradation products suggesting that that the bacteria were scavenging *n*-hexadecane as a carbon source and the long chain molecule was degraded step-by-step. Each and every degradation intermediates could not be detected in this work as the single solvent system may not be sufficient to elute all the different organic degradation products.

Microorganisms are well known to degrade C5–C16 alkanes and other common constituents of the crude petroleum (Jan et al., 2003). It was found that *P. aeruginosa* isolated from the polluted soil of a petroleum refinery can remove high doses of phenanthrene within 30 days (Romero et al., 1998; Haritash and Kaushik, 2009). The bacterium utilized in the present work is also a strain of *P. aeruginosa* and has been known to degrade crude oil sufficiently (77%) in a short period when the bioflocculating activity is the highest (Pathak et al., 2015). This may be due to the optimized production medium enriched with essential nutritional elements as compared to a minimal salt medium. From the data generated in this work, it is clearly seen that the bioflocculating activity is of a lower level in case of inoculated production medium with high molecular weight carbon sources. The ability to degrade low molecular-weight aromatic compounds, such as naphthalene, is widespread, and numerous reports have identified bacteria capable of utilizing these hydrocarbons for growth (Cerniglia, 1992; Sutherland et al., 1995). But the growth on aromatic substrates containing fused aromatic rings (e.g. chrysene, fluoranthene, pyrene, benz[a]anthracene) is somewhat rare, although organisms are known to use each of these aromatics as carbon source substrate.

The relationship between the rate of hydrocarbon degradation and the yield of bioflocculating activity is still doubtful but the results from this work definitely indicate that the bacterium, *P. aeruginosa*, shows the highest flocculating activity in presence of a single aliphatic hydrocarbon rather than a complex carbon source. For degradation, the EPS like bioflocculant could not be solely responsible, but yield of other compounds, such as emulcyan or rhamnolipids may be responsible for the enhancement of degradation *in vitro*. The degradation percentage of *n*-hexadecane by the subject bacterium was found to be higher than that of crude petroleum (77%) studied earlier (Pathak et al., 2015), because it is present as a single substrate nutrient instead of a complex mixture of nutrients composed of petroleum hydrocarbons, both long chain aliphatics and polyaromatics, when the bacterium needs much more energy to converting the complex substrate to a digestible one.

Contact angle measurement of the culture broth during *n*-hexadecane degradation has revealed some interesting results with respect to alkane biodegradation during the production of extracellular metabolites. This has been reported for the first time and could be considered as supportive data in respect of the degradation of the substrate. CA measurement was applied as a semi-quantitative analytical tool for measuring the hydrophilic behavior of a substrate undergoing biodegradation. It was found experimentally that the CA rapidly increased as *n*-hexadecane degradation rate went up and these changes could be represented as a direct measure of the degradation kinetics. The measurements revealed that the alcohols and acids formed during the biodegradation of *n*-hexadecane in the culture medium were polar and hydrophilic due to the hydroxyl end of the molecules. At the same time, the carbon chain is nonpolar and hydrophobic. As the carbon chain extends, the molecule becomes increasingly nonpolar and less soluble in water due to corresponding increase in hydrophobicity. The increase in bioflocculant production in the medium throughout the growth period was likely to be accompanied by a natural increase in the hydrophilic substances leading to an increase in the hydrophilic nature of the whole mass. As the moisture free glass surface is hydrophobic by nature, *n*-hexadecane droplet has the minimum CA on it (**Figure 2**).

Compositions of bioflocculants are often reported to be glycoprotein like substances where the conjugates are functional in showing flocculating activities (Sun et al., 2015a). In the present case, FTIR spectra became indicative of the presence of functional groups related to sugars and proteins. The FTIR analysis of the bioflocculant in this work showed the presence of –OH or –NH groups and the broad spectra appeared to be similar to that of a sugar-protein of complex origin. –OH, –COOH and COO– groups in the polymer molecules are important determinants for the flocculating activity while H^+^ and OH^-^ groups on the surface of the suspended particles may form hydrogen bonds when the bioflocculant chains approach the surface of particles.

A clear picture of the composition of the bioflocculant was obtained from the SEM-EDX results. Different bacteria involved in flocculation mostly produce EPS based bioflocculants. The present bacterium had produced the bioflocculant as an extracellular metabolite which had a composition consisting of the elements (C, N, or O) in a typical glycoprotein-like substance. Reports suggest that *Rhizobium* sp. and *Bacillus* sp. produce EPS mainly composed of C and O whereas a *Bacillus* sp. produces EPS that mainly comprises of N and O (Sun et al., 2015b). *Bacillus* species from marine environment had reportedly produced bioflocculant composed of C, N, O, P, and even S (Ugbenyen et al., 2014). The presence of Na, P, or Cl in the bioflocculant was primarily due to the attachment of elution buffer chemicals at the time of column purification (**Figure 4**).

Thermogravimetric analysis of the bioflocculant showed it to be composed of both partially thermolabile and thermostable molecules, a mixture of carbohydrates and proteinous substances as seen from the analysis. The first weight loss could be due to the loss of moisture content of the bioflocculant or of the protein part associated with glycoprotein-like molecules. Similar observations have been made by Wang et al. (2011) with respect to a bioflocculant obtained with mixed consortia of bacteria. In case of the bioflocculant, p-KG03, produced by a bacterial species, *Gyrodinium impudicum* KG03 (Yokoi et al., 1995), the initial weight loss was between 40 and 230°C with decomposition occurring at about 310°C. In the present work, the second decomposition occurred at 340–360°C. The stability of the bioflocculant produced by the *Pseudomonas* strain was up to 550°C that can be termed as moderately thermostable. The typical TGA curve of glucose in nitrogen environment also states the final decomposition within the range of 400°C (Zhishuang et al., 2014).

The ^1^H NMR could be confirmatory analytical tool in such investigations which revealed the presence of hydroxyl proton, non-anomeric protons and CH_2_ linkage in the sample, respectively. In the proton NMR, despite the fact that most resonances are often found clustered between 3.4 and 4.0 ppm, ^1^H spectra of carbohydrates also results some well-resolved signals related to those of anomeric protons (∼4.4–5.5 ppm), acetyl (∼ 2.0–2.1 ppm), and methyl (∼1.2 ppm) groups, and other protons which are influenced by specific functionality, including NH_2_ groups, phosphorylation, sulfation, glycosylation, and acetylation, or the lack of functionality as in deoxy-sugars (Bubb, 2003). However, possible shifts may be observed which may add plus or minus to the theoretical values due to secondary disturbances or nature of solvent utilized in analyses.

Production of EPS by bacteria is a well known phenomenon, but how EPS is involved right from signaling to utilization as building blocks in biofilm construction is yet to be fully understood. Determinable as a common entity, biofilms can be beneficial, for instance, to degrade environmental hazardous substances in the soil or in a bioreactor or as bioflocculants in the separation of particles from associated mineral matter like waste water (Bos et al., 1999). Moreover the biofilm matrix composed of EPS which also share a part in the composition of bioflocculants, often acts as an external digestion system by keeping extracellular enzymes close to biofilm cells, capable of metabolizing dissolved, colloidal and solid biopolymers. The EPS matrix constitutes the immediate environment and conditions of life for biofilm organisms and thus, is regarded as a key component for the understanding of the biofilm mode of life. Bacterial biofilms have been intensively studied not only because of their ecological importance, application in biotechnology, and waste water treatment but also due to their potential role in human infections and function as environmental reservoirs for pathogens (Jachlewski et al., 2015). However, a detailed composition analysis of an EPS content of bioflocculant tends to be difficult due to complex mixture of biomolecules and intermediates that act as functional and non-functional accessories. Furthermore, even though carbohydrates are identified as one of the major components of EPS, the biochemical properties of them remain elusive due to their complex structures and unique monomer linkages (Jiao et al., 2010). Characterization for determination of EPS monomers and even the associated proteins has been possible through HPLC, LCMS/MS, and LC/MS-ESI techniques (Stewart et al., 2013; Jachlewski et al., 2015). However, in bioflocculants the carbohydrate parts are seen profoundly present from other analyses. EPS is often implicated in the initial attachment of bacteria to surfaces and subsequent biofilm formation since it is present at the outermost layer of the cell (Yang et al., 2007). EPS has also emerged as a rich source of non-volatile sugar complexes, such as poly-*N*-acetyl glucosamine (PNAG), but that could be detected with GC/MS by proper derivatization with chemical agents like BSTFA (*N*,*O*-Bis trimethylsilyl) trifluoroacetamide), TMSCN (Trimethylsilyl cyanide) or MSTFA(*N*-methyl-*N*-(trimethylsilyl) trifluoroacetamide) (Wozniak et al., 2003; Khakimov et al., 2013). Alditol acetate derivatives of exopolysaccharides also presented successful determination of sugar monomers through GC/MS (Shi-Jie et al., 2011). In present work, GC/MS analyses of M-MSTFA derivatized bioflocculant detected EPS monomers as O-M-O-TMS derivatives. This analysis successfully identified all the sugar monomers associated with the composition of bioflocculant which were not completely understandable from the LC/MS analysis alone. This showed a preferable priority of GC/MS analysis in carbohydrate characterization upon ESI-LC/MS tool as it always forms different adducts from which the actual *m/z* were to be calculated without any confirmation from mass library.

Bacteria of different genera are known as bioflocculant producers and their application in heavy metal removal has received a lot of attention recently. In our study, the selectivity of the mentioned five heavy metals was based on by noticing the risking abundance of them in the samples chosen for isolation of bioflocculant producing bacteria which attained tolerance toward them as inborn nature. In a prior investigation (Subudhi et al., 2016) on heavy metal tolerant bacteria were screened for their bio-absorption of metals but the real application of bacterial cells are difficult in practical scenario, but it is easier if the effective metabolites responsible for heavy metal entrapment is used in practice and the outcome will be interesting. In the present study, the subject bioflocculant was able to flocculate the five mentioned heavy metal in a pattern of Ni^2+^ > Zn^2+^ > Cd^2+^ > Cu^2+^ > Pb^2+^. Initially the bioflocculant produced from *n*-hexadecane utilization was injected to 1 mg L^-1^ concentrations of the heavy metal amended aqueous solution, the pH of the whole content being unchanged in its optimum dose. The highest bioflocculating activity was found around pH 7 in this case. Heavy metal removal capacity of a bioflocculant in an aqueous system is reported to be lower at low and high pH values of the system; at low pH, a high concentration of protons competes for the anionic sites on the polymer preventing the removal of the divalent cations. Thus, divalent cation binding is low. This is actually the functional groups of any bioflocculant through which it acts to flocculation. Basically, the functional groups of EPS in bioflocculant are important factors determining their flocculation activity, especially when “bridging” could be considered as the most supported mechanism involved in flocculation. It is also found that the uronic acids in the bioflocculant help in the flocculation mechanism (Deng et al., 2005). In present case presence of glucuronic acid in the bioflocculant produced by this Pseudomonas species in confirmed through mass spectrometric analysis. Carboxyl groups present on the molecular chain of bioflocculants provide more sites for particle attachment and makes bridging between bioflocculant and discrete particles effective (Farooq et al., 2010). As the pH increases to its optimum value, which differ from one metal ion to another, the adsorbing surface is saturated with negative charges, resulted in increased efficiency to bind and adsorb metal cations. At pH higher than the optimum value, hydroxo species of the metals can be formed that do not bind to the adsorption sites on the surface of the adsorbent (Lin and Harichund, 2011). The stability of pH obtained in the present study could be advantageous in removing other heavy metals and pollutants too from various waste water streams by bioflocculants in required doses.

Bioflocculants derived from bacterial origin are reported to be of different structural origins varied with the source bacteria (Toeda and Kurane, 1991). In this case, in removing considerable amount of heavy metals from their aqueous solutions, the novel composition of the bioflocculant, a polymer of EPS and protein, could be instrumental. The main backbone of the EPS was composed of several sugars and proteins as the side chains which provided numerous flocculant binding sites, while the long backbone ensured the formation of large flocs (Shi-Jie et al., 2011). Supporting the involvement of the functional groups of related with bioflocculant, it was also evident from studies on Zn sorption on different bacterial strains which stated presence of acidic, neutral and basic group on the cell surface essential for bio-sorption (Guineä et al., 2006). The interactions between the exopolysaccharide based bioflocculant, and Ni^2+^, Zn^2+^ and other metal cations in aqueous solution leads to removal of the metal ions from water.

## Conclusion

This work, conducted with a selected microorganism, frequently designated as an opportunistic pathogen but which always bears a greater role in biosynthesis of molecules applicable for biodegradation of hydrocarbons, has revealed a few significant features with respect to the bioflocculant. Metabolites detected during substrate utilization (*n*-hexadecane) could be correlated with the production strategy of a novel bioflocculant, which could be described as a carbohydrate polymer capable of removing several heavy metals from water, with aerobic degradation pathway of aliphatic hydrocarbons. CA measurements have been established as a successful tool for investigating the interfacial behavior during the degradation process and subsequent exopolysaccharide-protein bioflocculant production may have immense possibilities for utilization in wastewater treatment.

## Author Contributions

MP as Ph.D. research scholar had taken part in all laboratory scale experiments, analysis as well as writing the manuscript. AD and HS had created the basic research outline. KB had supported in writing and research planning from his professional experience. The collaborators SS, VB, and experts like BL had contributed in laboratory works, writing and revising the manuscript. However, all the authors had gone through the manuscript and revised it technically before submission.

## Conflict of Interest Statement

The authors declare that the research was conducted in the absence of any commercial or financial relationships that could be construed as a potential conflict of interest.
